# Trajectories of Activities of Daily Living for Patients with Advanced Cancer Beyond the Last Eight Weeks of Life. Implications for Palliative Rehabilitation

**DOI:** 10.1089/pmr.2024.0034

**Published:** 2024-07-19

**Authors:** Deidre D. Morgan, Jennifer J. Tieman, Magnus P. Ekström, David C. Currow

**Affiliations:** ^1^ Research Centre for Palliative Care, Death and Dying (RePaDD), College of Nursing and Health Sciences, Flinders University, Bedford Park, Australia.; ^2^ Faculty of Medicine, Department of Clinical Sciences Lund, Respiratory Medicine and Allergology, Lund University, Lund, Sweden.; ^3^ Graduate School of Medicine, Faculty of Science, Medicine and Health, University of Wollongong, Wollongong, Australia.

##  

Soeda et al.^1^ are to be commended for their retrospective study on Activities of Daily Living trajectories of people with advanced cancer in the past eight weeks of life.^1^ We agree there is an imperative to understand characteristics of functional decline to inform tailored patient care. This study highlights the absence of trajectory studies that enable identification of gradual functional changes and used the functional independence measure (FIM) to measure functional decline in their study.

An earlier study published in *Palliative Medicine*^2^ about trajectories of functional decline measured prospectively over the past four months of life (*n* = 55,548) used the Australia-modified Karnofsky Performance Status (AKPS) scale^3^ to measure function or Activities of Daily Living abilities. We identified only two trajectories of functional decline:

- Trajectory 1: Cancer, solid organ failure, and cardiovascular disease (requiring sustained moderate assistance with Activities of Daily Living); and
- Trajectory 2: Dementias and other neurological conditions (requiring sustained maximal assistance with Activities of Daily Living).

Although requiring different levels of functional assistance over time, there was a rapid deterioration in function for both trajectory cohorts in the last two weeks of life. (Fig. 1). This is consistent with the “Rapid decline” described by Soeda et al. (*n* = 43).^1^

**FIG. 1.**
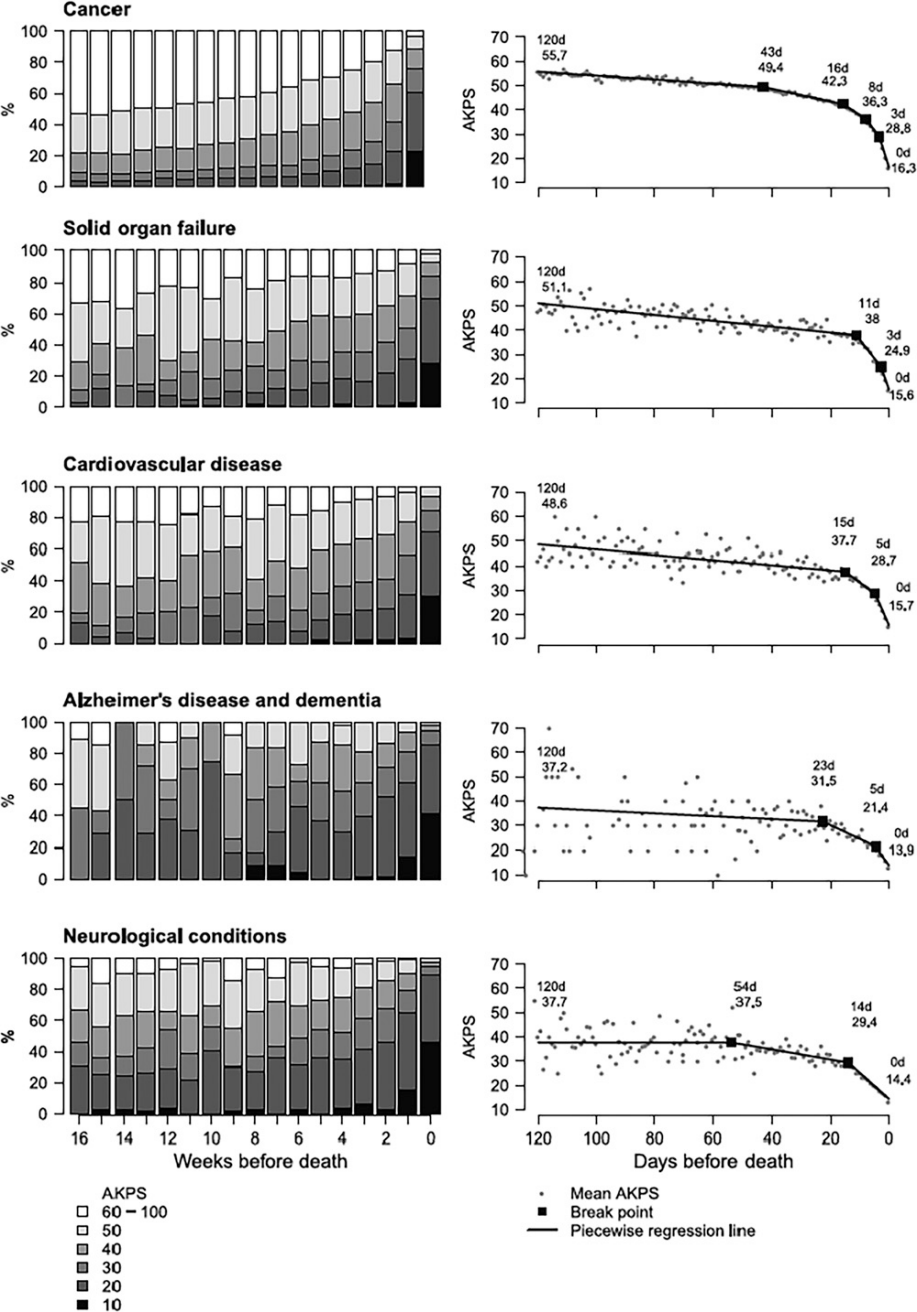
Distribution of weekly AKPS scores prior to death (Morgan et al.^2^). AKPS, Australia-modified Karnofsky Performance Status.

Recommendations about the importance of identifying patient levels of independence and levels of graded assistance required are welcomed. Morgan et al.^2^ also demonstrated that people with cancer (*n* = 39,783) had a sustained AKPS score of between 60–50 (120 days–43 days of death) where they required minimum to moderate assistance with activities of daily living. This is a time where patients and their carers could particularly benefit from interventions to optimize function. These findings are similar to the no decline, rapid decline and moderate disability, and slow decline trajectories described by Soeda et al.^1^ Importantly, the AKPS, used by Morgan et al.^2^ can be conducted by any health professional with minimal training, is quick to administer and can be used as a screening tool to guide referral to rehabilitation professionals such as occupational therapists and physiotherapists (e.g., an AKPS score of ≤60 warrants referral to allied health to optimize function). By contrast, the FIM takes 30–45 minutes to complete and assessors must be credentialed and recredentialed every two years at cost. The AKPS may be an acceptable alternative for the majority of clinical settings with explicit criteria for referral to rehabilitation teams.

Better understanding of functional trajectories can inform the types of services required to provide optimal care. However, we note that although rehabilitation professionals such as occupational therapists, physiotherapists are skilled in assessing and implementing interventions to optimize function at the end-of-life, they were not named in recommendations by Soeda et al.^1^ As patients live longer with life-limiting illnesses, we must consider adopting a rehabilitative approach to optimize function at every stage of disease progression. This is consistent with the World Health Organization’s recent briefing^4^ on integrating rehabilitation into palliative care. Considered through this lens, rehabilitation professionals are pivotal members of palliative care teams.

## Abbreviations Used

AKPS: Australia-modified Karnofsky Performance Status Scale
FIM: Functional Independence Measure
